# Endothelial dysfunction in autoimmune, pulmonary, and kidney systems, and exercise tolerance following SARS-CoV-2 infection

**DOI:** 10.3389/fmed.2023.1197061

**Published:** 2023-07-27

**Authors:** Sabyasachi Sen, Shikha Khosla, Omar Awan, Scott Cohen, Jared M. Gollie

**Affiliations:** ^1^Division of Endocrinology, Department of Medicine, Veterans Affairs Medical Center, Washington, DC, United States; ^2^Division of Endocrinology, Department of Medicine, George Washington University, Washington, DC, United States; ^3^Division of Pulmonary Medicine, Department of Medicine, Veterans Affairs Medical Center, Washington, DC, United States; ^4^Division of Pulmonary, Critical Care, and Sleep Disorders Medicine, The George Washington University, Washington, DC, United States; ^5^Division of Nephrology, Department of Medicine, Veterans Affairs Medical Center, Washington, DC, United States; ^6^Research and Development Service, Veterans Affairs Medical Center, Washington, DC, United States; ^7^Department of Health, Human Function, and Rehabilitation Sciences, The George Washington University, Washington, DC, United States

**Keywords:** post-acute and long COVID symptoms, endocrine disorders, endothelial (dys)function, A-vO2 difference, kidney insufficiency, diffusion capacity (DLco)

## Abstract

Long COVID is characterized by persistent symptoms beyond 3-months of severe acute respiratory syndrome Coronavirus-2 (SARS-CoV-2) infection that last for at least 2 months and cannot be explained by an alternative diagnosis. Autonomic, immunologic, endothelial, and hypercoagulation are implicated as possible mechanisms of long COVID symptoms. Despite recognition of the public health challenges posed by long COVID, the current understanding of the pathophysiological underpinnings is still evolving. In this narrative review, we explore the long-term effects of SARS-CoV-2 infection on T cell activation such as autoimmune disorders and endothelial cell dysfunction involving vascular impairments within pulmonary and renal architecture. We have described how endothelial dysfunction and vascular abnormalities may underscore findings of exercise intolerance by way of impaired peripheral oxygen extraction in individuals with long COVID.

## 1. Introduction

Severe acute respiratory syndrome Coronavirus-2 (SARS-CoV-2) poses an ongoing public health challenge with the long-term after-effects yet to be fully appreciated. According to the Center of Disease Control’s (CDC’s) coronavirus disease (COVID-19) data tracker, 6,183,075 total hospitalizations and 1,132,206 deaths have resulted due to COVID-19 complications in the United States (US) between January 2020 and May 2023 (1). The clinical presentation following SARS-CoV-2 infection ranges from mild or asymptomatic to severe or critical with respiratory distress, cytokine storm, and coagulopathy. Certain chronic comorbidities, such as hypertension, cardiovascular disease, obesity, diabetes, and kidney disease, are highly prevalent in people with COVID-19. While these comorbidities do not appear to increase the risk of developing COVID-19, they are associated with an increased risk of disease severity and mortality (2). Several studies over the course of the pandemic have reported that COVID-19 is associated with hyperglycemia in people with and without known diabetes (3, 4). Moreover, a number of studies have reported new-onset diabetes as being associated with the presence of COVID-19 and current data also suggest a bidirectional relationship between type 2 diabetes mellitus (T2DM) and COVID-19 (5).

SARS-CoV-2 is an enveloped positive strand RNA(+ssRNA) virus which has a ~ 30 kb RNA genome. The 3′-terminus of this genome encodes four structural proteins including the spike surface glycoprotein (S) which is composed of peripheral (S1) and transmembrane (S2) subunits (6). The SARS-CoV-2 virus gains access to host cells through the angiotensin-converting enzyme 2 (*ACE2*) receptor. Data suggests that the hosts genetic makeup influences the susceptibility, immune response, and outcomes to SARS-CoV-2 infection (7). In their review, Fricke-Galindo et al. (7) noted that HLA-A*25:01, -B*15:27, -B*46:01, -C*01:02, and-C*07:29 alleles are associated with COVID-19 susceptibility; while HLA-A*02:02, -B*15:03, and-C*12:03 are alleles with low risk of infection. Similarly, variants in cytokine genes like *IL1B*, *IL1R1*, *IL1RN*, *IL6*, *IL17A*, *FCGR2A*, and *TNF* could be associated with disease susceptibility and cytokine storm, and/or COVID-19 complications (7).

The virus spike protein (composed of S1 and S2 subunit), protrudes from the virus surface and binds to ACE2 receptor. After binding to the *ACE2*, the S1 subunit dissociates with the *ACE2* receptor, through a process that requires the transmembrane serine protease 2 (*TMPRSS2*) protein. The resultant conformational change allows the S2 subunit to fuse with the host cell membrane. Unlike other coronaviruses, SARS-CoV-2 does not appear to use other receptors for cellular access and binding to the *ACE2* receptor is obligatory for SARS-CoV-2 cellular entry (8). In humans, both *ACE2* mRNA and *TMPRSS2* mRNA are expressed in several endocrine glands, including the pancreas, thyroid gland, ovaries, and testes. This makes these endocrine glands particularly susceptible to both destruction and dysfunction from COVID-19 infection.

Our understanding regarding the pathogenesis of inflammation has evolved from evidence on the acute local infection with systemic inflammatory response to the chronic low intensity inflammation induced by the SARS-CoV-2 virus (9). The four stages of invasion described by Gusev et al. (9) are as follows:

1. Primary blockage of the innate immunity: The invasion stage involves the attachment of the Spike protein to the *ACE2* receptors and co-receptors and alternate receptors (as described above). After incorporation into the cell, as SARS-CoV-2 multiplies, it also blocks off mechanisms of innate immunity.
2. Virus protection against adaptive immunity that involves the T and B lymphocyte activation
3. Acute complications secondary to resultant inflammation
4. Long term complications as a progression from acute ones

**Figure 1:**
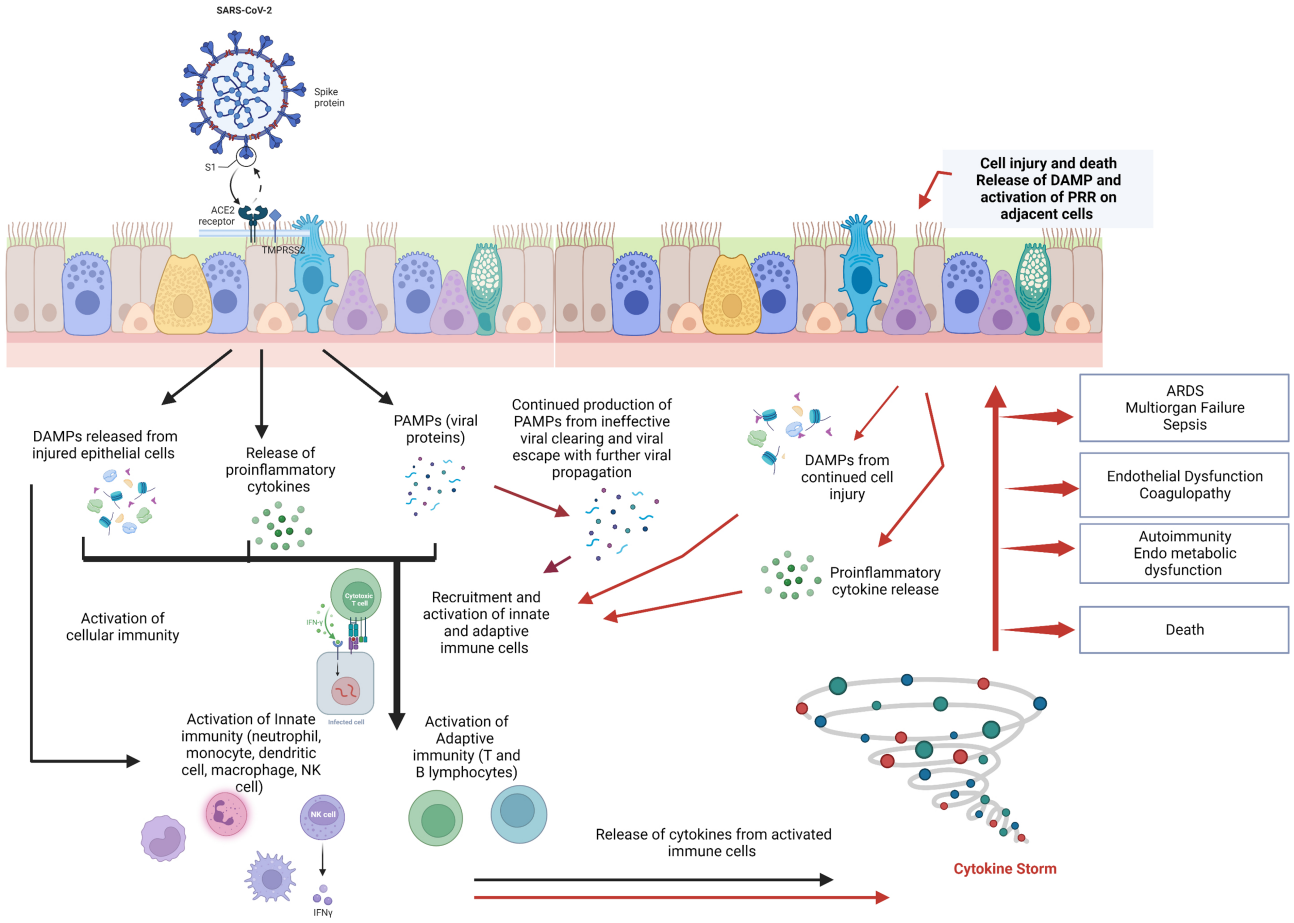
SARS-CoV-2 spike protein binds to ACE2 receptor of the host’s cells, primarily the pulmonary epithelial cells. This stimulates the release of PAMPs, DAMPs, and cytokines/chemokines into the cellular microenvironment and results in the recruitment and activation of innate immune cells followed by adaptive immune cells to the site of damage. In cases with normal physiologic immune cascade activation, the immune response effectively clears the virus, thus resolving infection and restoring tissue homeostasis. However, in patients with immune dysregulation, there is a hyper-inflammatory response (the “cytokine storm”), with further damage to the pulmonary epithelium in a positive feedback loop but ineffective viral clearing. Damaged epithelial cells stimulate the release of more pro-inflammatory chemokines/cytokines and DAMPs, exacerbating epithelial cell damage and death. Cytokine storm syndrome causes of acute respiratory distress syndrome (ARDS), endothelial dysfunction, sepsis, and multiple-organ dysfunction. Endothelial dysfunction results in coagulation cascade activation resulting in microthrombi and further complicating the clinical picture [Figure adapted from Burgoyne et al. (10)]. Created with BioRender.com.

The SARS-CoV-2 virus penetrates target cells *via* various cell surface receptors, such as *ACE2* and *TMPRSS2*. While certain receptors (like *ACE2*) and co-receptors/cofactors (like Neuropilin 1) allow cell entry while other receptors (called pattern recognition receptors) initiate an anti-viral immune response. This pattern recognition receptors (PRRs) recognize components of the attacking virus, or the cell components of damaged/dying host cells known as pathogen associated molecular patterns (PAMP) or Damage-associated molecular patterns (DAMP) respectively (9). Once these PRRs are activated, they activate innate immunity that includes antiviral action of Interferons (IFNs), complement system macrophages, neutrophils, mast cells, natural killer (NK) cells and the coagulation system (see Figure 1). Activation of all these pathways results in rise in body temperature, drop in neutrophil, red cell and platelet counts, changes in vascular permeability, microthrombi formation, inflammation, oxidative stress and elevation of ferritin, fibrinogen, and D-dimer.

Destruction of cells lead to recruitment of additional macrophages and monocytes that release cytokines and activate the adaptive immunity of T and B cells (11). In most cases, the host immune response overcomes the inhibitory effects exerted by the virus and is able to clear the pathogen from the body. However, in certain susceptible populations, namely those 80 years of age and older, multiple existing comorbidities, and immunocompromised, the SARS-CoV-2 exerts its pathogenicity and inhibits innate immunity. It also disrupts specific immune pathways as well as universal cell distress signaling pathways. This protects the virus and provokes a dysfunctional immune system to attack host tissues via autoimmune and autoinflammatory processes. In the acute phase, with the excessive release of cytokines (“cytokine storm”), a severe systemic inflammatory response with associated complications can occur. Wang et al. (12) used a high-throughput autoantibody discovery method known as rapid extracellular antigen profiling (REAP) in 194 patients and demonstrated high levels of antibodies against immunomodulatory proteins (cytokines, complement system, chemokines etc.) as compared to uninfected controls. These antibodies ranged from wide spectrum antibodies (like antinuclear antibodies, antiphospholipid antibodies, anticytoplasmic antibodies) to specific antibodies (like anti IFN alpha antibodies, antiglutamic acid decarboxylase antibodies, antithyroglobulin antibodies).

With immune system dysfunction, viral clearing gets delayed triggering chronic inflammation promoting persistent symptoms of COVID-19 (9, 11). Persistence of antinuclear antibody titers ≥1:160 in 43.6% of 142 patients at 12 months post–COVID-19 symptom onset have been described in the literature (13, 14). Similar to other post-acute viral syndromes, there are increasing reports of persistent and prolonged effects after acute COVID-19 termed long COVID (also referred to as post-acute sequelae COVID-19, post-COVID syndrome, and long haulers) (15–17). The clinical case definition of long COVID, as defined by the World Health Organization (WHO), includes individuals with a history of probable or confirmed SARS-CoV-2 infection, usually 3 months from the onset of COVID-19 with symptoms and that last for at least 2 months and cannot be explained by an alternative diagnosis (18). Long COVID has been described by some as ongoing constitutional symptoms of fatigue, dyspnea, cognitive impairment, mood alterations, headaches, joint and chest pains, muscle aches, cough, smell and taste dysfunction that persist for over 4 weeks after symptom onset or hospital discharge (19). Using the Long COVID Symptom Tool, 85% of symptomatic patients after 2 months were still reporting symptoms 1 year following symptom onset (20). Importantly, reductions in quality of life were reported to increase 6 months after the onset of symptoms (20).

Vaccine-induced immunity, natural immunity, and the combination of the two (i.e., hybrid immunity), offer protection against hospitalization and mortality from SARS-CoV-2 infection (21–24). Based on low level evidence, vaccination prior to infection and after infection is shown to reduce the incidence of long COVID, with two doses being more beneficial than one dose (25, 26). Additionally, vaccinating individuals with existing long COVID symptoms does not seem to offer beneficial effects on symptomatic reduction (27). In individuals with breakthrough infections, the risk of long COVID is lower when compared to those with SARS-CoV-2 infection without prior vaccination (28). Thus, vaccination prior to infection seems to provide some protection against the development of long COVID but does not eliminate the risk altogether. High quality data is needed to determine how potential confounding factors influence the impact of vaccination status on long COVID. Moreover, data on effects of natural and hybrid immunity are currently limited and require further investigation.

The prevalence of long COVID is reported to range between 10 to 70% and higher in those infected by the historical variant when compared to Alpha, Delta, or Omicron (29, 30). Female sex, pulmonary disease, diabetes, obesity, and organ transplantation have been identified as potential risk factors for long COVID (31). Of note, autonomic, immunologic, endothelial, and hypercoagulation are implicated as possible mechanisms underlying long COVID (32). Here, in this narrative review, we aim to extend the current understanding of long COVID by exploring the long-term effects of SARS-CoV-2 infection on T cell activation such as autoimmune disorders and endothelial cell dysfunction involving vascular impairments within pulmonary and renal architecture. We then describe how endothelial dysfunction and vascular abnormalities may underscore findings of exercise intolerance by way of impaired peripheral oxygen extraction in individuals with long COVID. Comprehensive literature searches of PubMed/MEDLINE, Web of Science, Google Scholar, and select reference lists were conducted from inception to May 2023 using the following primary search terms: SARS-CoV-2, COVID-19, Post-acute Sequelae of COVID-19, Long COVID.

## 2. SARS-CoV-2 infection and autoimmune endocrine disorders

### 2.1. Thyroid disease

It is well known that thyroid hormones modulate innate and adaptive immune responses through both genomic and nongenomic mechanisms. Physiological concentrations of L-thyroxine (T4) and 3,3′,5-triiodo-L-thyronine (T3) stimulate the production and release of cytokines, which are also components of “cytokine storm” in viral infections. Also, T4 and T3 can potentiate the IFN. The immune cascades stimulated (i.e., the cytokine and hyperactivation of Th1 helper cells response) in response to viral infection are the same as those observed in autoimmune thyroid disorders (AITD), and IFN related thyroid disease (33). Patients can present with hyperthyroidism due to subacute thyroiditis (SAT), painless thyroiditis, thyroid hormone toxicosis or Grave’s disease; or hypothyroidism from primary or central hypothyroidism; or euthyroid sick syndrome. Out of these thyroid derangements, SAT appears to be most common in acute COVID settings similar to several other viral infections (34).

Using a retrospective analysis, Bostan et al. found that the incidence of newly diagnosed SAT was 0.136% in 2018, 0.127% in 2019, 0.157% in 2020, and 0.114% in 2021 (*p* = 0.19). They also noted that in their cohort, SAT patients were clustered in the autumn (35.1%) in 2018 and 2019, and that this cluster shifted to the winter (33.0%) in 2020 and 2021, in parallel with COVID-19 case peaks (35). SAT can occur during acute COVID-19 infection or typically within 6–8 weeks after the viral infection. It occurs most commonly in middle-aged females and the clinical presentation can be varied. Some data suggests that neck pain is more intense, fever more frequent, and post SAT hypothyroidism is more common than other forms of SAT. Importantly, post-COVID-19 fatigue may be due to residual post-SAT hypothyroidism. Treatment of SAT requires the same approach as non-COVID-19 SAT with use of non-steroidal and glucocorticoid therapy (36). Similarly, management of all other thyroid conditions follows current established practice guidelines for these conditions.

### 2.2. Adrenal disease

There are several putative mechanisms by which SARS-CoV-2 can impact adrenal function. These include hypothalamic–pituitary–adrenal (HPA) axis dysfunction with critical illness-related corticosteroid insufficiency; direct cytopathic impact of the virus on the adrenals, pituitary, and hypothalamus; immune-mediated inflammation; small vessel vasculitis; microthrombotic events; resistance of cortisol receptors; and impaired post-receptor signaling; and dissociation of adrenocorticotropic hormone and cortisol regulation (37). Hyponatremia is a common finding in acute COVID-19 infection and serum sodium levels <135 mmol/L may be associated with poorer outcomes (38). It could result from syndrome of inappropriate antidiuretic hormone secretion, hypovolemia as well as adrenal insufficiency. Several case reports of adrenal infarction and adrenal hemorrhage have been described in the literature, in acute COVID settings (39).

### 2.3. Disorders of the pancreas

Wu et al. (40) mechanistically linked acute COVID-19 to diabetes and found that the SARS-CoV-2 receptor, *ACE2*, and related entry factors (*TMPRSS2*, Neuropilin 1, and Transferrin receptor) are expressed in beta cells, with selectively high expression of Neuropilin 1 (NRP1). They showed that SARS-CoV-2 infected human pancreatic beta cells in patients who died of COVID-19 and selectively infected human islet beta cells *in vitro*. Their *ex-vivo* and *in vitro* studies showed that SARS-CoV-2 directly induced beta cell death by apoptotic beta cell signaling, similar to that observed in type 1 diabetes (T1DM). Proposed mechanisms include direct virus-mediated injury, systemic inflammatory response as well as circulating proinflammatory interleukins, lipotoxicity induced by the virus, and drug-induced injury (41).

Hyperglycemia is a common occurrence in acute COVID-19 infections and has been widely reported even in patients not previously known to be diabetic (42). Several reports of ketosis, new onset T1DM as well as T2DM have been published in the literature (42). Other reports have indicated that hyperglycemia upregulated *ACE2* and *TMRSS2* cell surface receptors thereby augmenting possibility of infection with COVID-19. Sathish et al. conducted a meta-analysis and showed a pooled proportion of 14.4% for newly diagnosed diabetes in hospitalized COVID-19 patients (43). It is widely accepted that patients with sub-optimally controlled diabetes have a more severe and protracted course of COVID-19 illness and poorer outcomes. The complex interplay between SARS-CoV-2 and pancreas/diabetes in acute and long COVID continues to be an area of extensive research worldwide (44).

### 2.4. Disorders of the gonads

Men with acute COVID-19 have been reported to have high levels of prolactin and luteinizing hormone and low levels of testosterone and follicle-stimulating hormones, indicating possible primary testicular damage during active disease (45). Studies have shown that *ACE2* expression is observed in seminiferous tubules, Leydig cells, and Sertoli cells (46). It plays an important role in testosterone or steroidogenesis regulation, interstitial fluid volume, and in maintaining healthy spermatogenesis (46). The underlying mechanism of SARS-CoV-2 action on the male gonads remains unclear but possible actions include infection-induced oxidative stress, HPA axis dysfunction due to acute severe infection, and direct gonadal damage (46, 47). Concerns regarding long term spermatogenic failure, sperm alterations, and male infertility are still areas of uncertainty. In their review of 148 published papers on impact of COVID on fertility, Ata et al. (48) noted that there appears to be no co-expression of *ACE2* and *TMPRSS2* in the myometrium, uterus, ovaries, or fallopian tubes. Oocytes on the other hand may be susceptible to SARS-CoV-2 infection due to presence of the *ACE2* receptor and *TMPRSS2* coreceptor; however, viral RNA in oocytes has not been reported thus far. Embryos, especially late blastocysts, may be susceptible to SARS-CoV-2 infection. Most studies have not reported a significant impact of COVID-19 on ovarian reserve, ovarian function, or follicular fluid parameters. Transient impact of COVID-19 on menstrual patterns may occur (48). Further studies are needed to advance our understanding on impact of COVID on female fertility.

Most of the endocrinopathies described herein pertains to acute COVID setting and research is ongoing in the long COVID setting to decipher which of these conditions can persist long-term.

## 3. SARS-CoV-2 infection, lung function, and pulmonary vasculature

The mortality of acute COVID is often determined by the severity of respiratory failure. Significant endothelial dysfunction and coagulopathy have been noted in acute COVID pathogenesis (49). Autopsy studies have found progressive diffuse alveolar damage, but also significant pulmonary arterial thromboses (50, 51). Thus, it is thought that endothelial dysfunction is a critical component of acute pulmonary pathogenesis including progressive hypoxic respiratory failure and acute respiratory distress (52). Moreover, there are suggestions that endothelial dysfunction may contribute to ongoing symptoms in long COVID.

An early meta-analysis indicated that 24% of people survive their acute COVID episode continue to have unresolving dyspnea (53). This meta-analysis did not stratify patients by acute severity, however in other studies, prevalence of dyspnea appeared to correlate with severity of infection with outpatients having the least persistent dyspnea and critically ill patients having the most (54). In those with residual pulmonary fibrosis, the cause of dyspnea is clear. Still, many patients have persistent dyspnea without any significant CT abnormalities. Especially in those with mild COVID who never developed hypoxic respiratory failure, pulmonary fibrosis is rarely seen, though dyspnea is often reported (55). There are numerous hypothesized mechanisms for dyspnea in long COVID, of which endothelial dysfunction is thought to represent one possibility. Causes other than pulmonary fibrosis includes chronic pulmonary emboli, worsening of underlying COPD or asthma, diaphragmatic dysfunction, and potentially a poorly described neurologic phenomenon of mismatched respiratory effort with respiratory sensory inputs, which could be from either a peripheral neuropathy or central neurologic change.

On pulmonary function testing, the most common abnormality is an impairment in diffusing capacity (56). Diffusing capacity of carbon monoxide (DLCO) is a measure of the ability of gas to transfer from the lungs to the red blood cells, by traversing the alveoli, basement membrane, and pulmonary capillaries (57). Limitations in any of those areas such as anemia, interstitial lung disease or emphysema, and pulmonary vascular disease can result in a decreased diffusing capacity. Kersten et al. (58) demonstrated that individuals with highly symptomatic long COVID experienced impaired diffusion capacity and reduced distance in the 6 min walk test despite having average or only mildly affected mechanical lung function. While the exact mechanisms explaining impaired diffusion capacity in long COVID is unclear, when noted in those with a relative lack of pulmonary fibrosis or emphysema, it strongly suggests a component of pulmonary vascular disease or potentially anemia. Early data on patients discharging from the hospital indicated that anemia and deconditioning were significant factors for cardiopulmonary limitations (59). However, a recent meta-analysis of studies utilizing CPETs reported numerous possibilities beyond anemia or deconditioning, including decreased peripheral oxygen extraction, preload failure, chronotropic incompetence, and various pulmonary limitations (60). Thus, there are many hints that the prolonged respiratory difficulties experienced by those who survive SARS-CoV-2 infection may be related to a myriad of vascular issues.

SARS-CoV-2 results in reduced *ACE2* during the recovery period, which may result in a slight hypercoagulable state. Furthermore, persistent SARS-CoV-2 spike protein has been reported in long COVID patients, which may promote a prolonged inflammatory response (61). The combination of reduced *ACE2* and persistent inflammatory response may lead to chronic pulmonary vessel remodeling, though remodeling in other areas likely affects pulmonary function. While some cases of dyspnea may be due to direct damage to the pulmonary vasculature (even in the absence of pulmonary fibrosis), others may be due to increased venous capacitance resulting in the decreased preload with exercise. And finally, there may be decreased peripheral oxygen extraction, which may be a vascular/capillary phenomenon resulting in poor oxygen delivery to tissues and essentially left to right shunting.

## 4. SARS-CoV-2 and kidney disease

Acute Kidney Injury (AKI) is common in the setting of active COVID-19 infection. More than 20% of patients who are hospitalized with COVID-19 infection develop AKI and the numbers are even higher, approaching 50% in patients who are admitted to the ICU (62–65). Nearly 10% of all hospitalized patients with COVID-19 infection require kidney replacement therapy. AKI is a significant risk factor for chronic kidney disease (CKD) (65, 66). In a retrospective observational cohort study of 12,891 hospitalized adult patients with prior SARS-CoV-2 infection, it was found that an episode of COVID-19 associated AKI was associated with decreased survival (67). This hospitalized cohort of COVID-19 patients had a particularly high rate of AKI with more than 50% of the patients having a minimum of one episode of AKI. The severity of the AKI episode was also associated with decreased recovery of kidney function and increased mortality.

Al-Aly et al. (68) evaluated the electronic health records from the Veterans Health Administration and found that SARS-CoV-2 infection increased the long-term risk of developing CKD and that the risk was particularly high in those with the most severe cases of viral infection. Among hospitalized Veterans with COVID-19 infection, there was incident development of AKI and CKD after 30 days of a COVID-19 infection. Likewise, a study from China showed 35% of patients had an eGFR <90 mL/min/1.73 m^2^ 6 months after being hospitalized with a diagnosis of SARS-CoV-2 infection (69). Of the patients who had normal kidney function initially, 13% subsequently developed a decrease in estimated glomerular filtration rate (eGFR) at follow-up.

The pathogenesis of AKI in acute COVID-19 infection is multifactorial and involves activation of the immune system, coagulation cascade, endothelial injury, and the renin-angiotensin-aldosterone system. Hypotension, low cardiac output, development of renal microthrombi, nephrotoxic medications and hypoxia may also contribute. Acute renal tubular injury is the most common histopathology seen in COVID-19 associated AKI. There are also reports of glomerular injury with thrombotic microangiopathy and collapsing focal segmental glomerulosclerosis (70).

COVID-19 infection may impact the kidney through direct effects of the virus or indirectly through the systemic inflammatory response (65). It is unclear if the virus directly invades kidney cells as there is no evidence of viral replication using molecular techniques including RNA *in situ* hybridization and immunohistochemistry. However, positive COVID-19 polymerase chain reaction (PCR) tests of kidney tissue have been documented (71). Nandula et al. has shown in a small group of pre-diabetic post-COVID patients that podocytes may be affected in the kidneys and podocyte specific proteins may remain elevated even up to 1 year post infection (72). Interestingly, podocyte specific protein estimation in the urine has been established as an early and sensitive biomarker for cardio-renal function in those with diabetes and prediabetes (73).

SARS-CoV-2 viral effects may persist long after clinical resolution of the infection. The immune system’s response to the virus could potentially trigger increased predisposition to recurrent AKI and thus increased chances of CKD development (74). However, the long-term pro-inflammatory impact of a prior COVID-19 infection requires further study. It is also possible that the COVID-19 infection increases the risk for incident or worsening of preexisting diabetes mellitus, hypertension, and cardiovascular disease thus leading to increased risk for CKD.

Progressive CKD in the setting of SARS-CoV-2 infection likely involves multiple mechanisms including persistent inflammation and maladaptive repair of injured nephrons from prior AKI. There can be resultant compensatory hypertrophy of remaining nephrons. These mechanisms can come together potentially leading to interstitial fibrosis and glomerulosclerosis. However, not all patients with SARS-CoV-2 infection and prior AKI develop progressive CKD. There are different trajectories of kidney function following SARS-CoV-2 infection including patients who develop rapid decline in kidney function following viral infection, complete recovery, recurrent AKI, incomplete recovery of kidney function with no further decrease in GFR, and incomplete recovery of kidney function with progressive CKD (74). Therefore, the interaction of SARS-CoV-2 infection with other predisposing genetic risk factors for progressive CKD including *APOL1* gene mutations needs to be explored further (75).

## 5. Exercise intolerance

**Figure 2:**
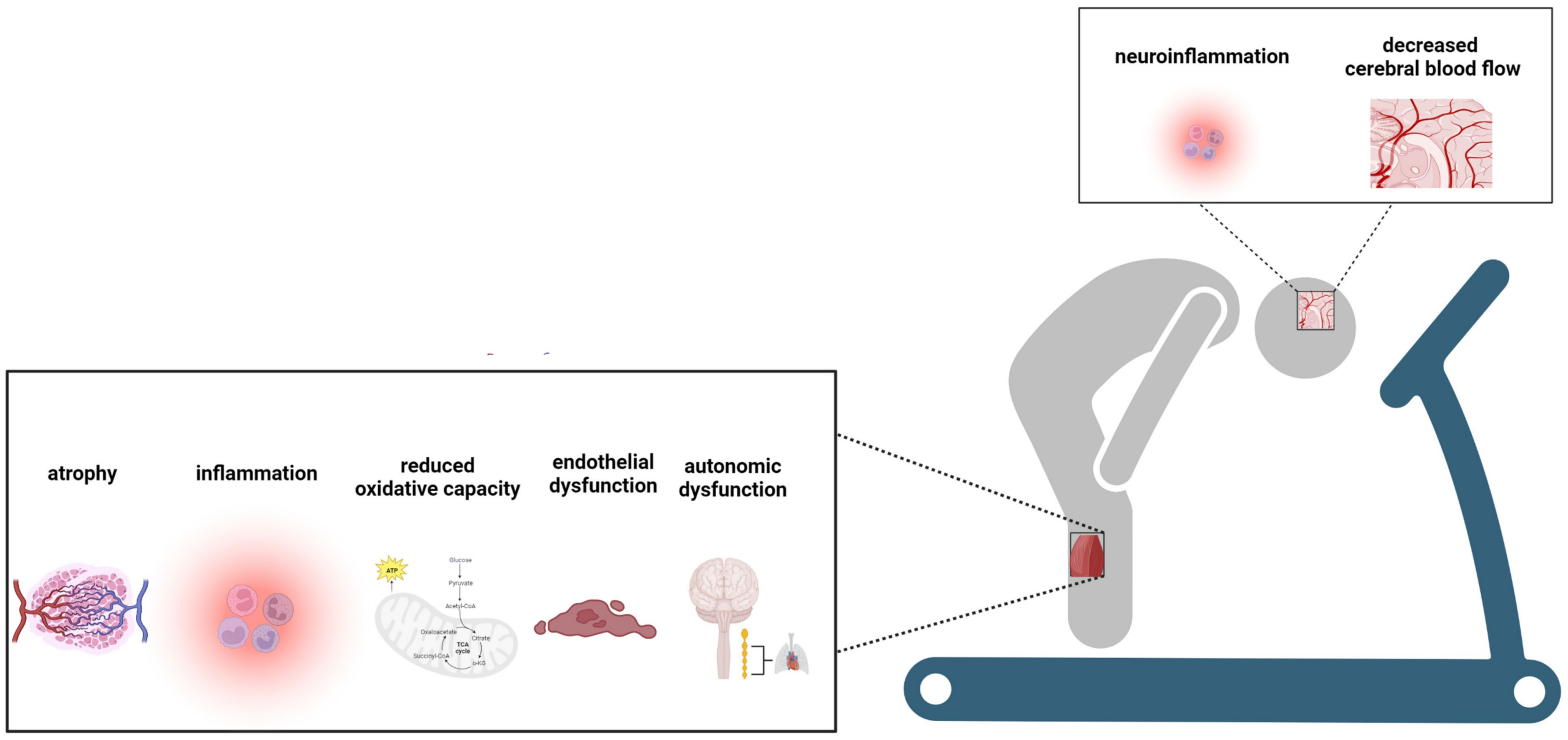
Potential mechanisms of exercise intolerance to exercise intolerance in individuals following SARS-CoV-2 infection. Created with BioRender.com.

Individuals living with long COVID often experience reductions in exercise capacity (i.e., exercise intolerance). In a meta-analysis of 9 studies including 464 individuals with long COVID symptoms and 359 without symptoms who completed CPET assessments, mean peak oxygen consumption (VO_2_) was found to be 4.9 mL/kg/min lower in those with long COVID symptoms compared to those without symptoms (60). Several potential sites involved in the oxygen delivery and utilization pathway have been implicated in exercise intolerance in individuals previously infected with SARS-COV-2 and those experiencing long COVID (59, 60, 76–84). Of note, impaired peripheral oxygen extraction has surfaced as one potential site of importance when considering limiting factors contributing to exercise intolerance in long COVID (see Figure 2) (59, 60, 78, 82). Singh et al. (78) compared systemic and pulmonary hemodynamics, ventilation, and gas-exchange in 10 patients who recovered from COVID-19 and without cardiopulmonary disease to 10 age-and sex-matched control participants. Invasive CPET examinations revealed reductions in peak VO_2_ to be associated with impaired systemic oxygen extraction despite preserved peak cardiac index (78).

Peripheral oxygen extraction is dependent on hematocrit level, kinetics of oxygen off-loading from hemoglobin, erythrocyte mean capillary transit time, diffusional oxygen conductance, capillary density, and muscle oxidative capacity (85). Reductions in peripheral oxygen extraction have been reported in clinical populations with metabolic myopathies (86). Skeletal muscle alterations including reduced force capacity, fiber atrophy, mitochondria and metabolic dysfunction, and capillary impairments have all been observed in patients following SARS-CoV-2 infection and are thus likely to contribute to reduced peripheral oxygen extraction in those with long COVID (87). Additionally, endothelial and autonomic dysfunction due to SARS-CoV-2 infection may decrease erythrocyte mean capillary transit time due to vasoconstriction. Future studies are needed to confirm the contributions of these factors to reduced peripheral oxygen extraction and exercise intolerance in long COVID.

## 6. Physical activity and long COVID

Physical activity level is a strong predictor of adverse outcomes following SARS-CoV-2 infection (88, 89). Those who are consistently inactive have greater risks for hospitalization, admission to the ICU, and death due to COVID-19 than individuals who are doing some physical activity or consistently meet the physical activity guidelines (88). These findings underscore the potential importance of physical activity and fitness for combatting outcomes resulting from SARS-CoV-2 infection and has led to the proposal of various interventions including exercise, cardiopulmonary rehabilitation, diaphragmatic breathing techniques, cognitive behavioral therapy, and mindfulness training (90–95). In a systematic review, rehabilitation seemed to improve dyspnea, anxiety, kinesiophobia, muscle strength, walking capacity, sit-to-stand performance, and quality of life (96).

## 7. Post-exertional symptom exacerbation and exercise in long COVID

Despite promising preliminary findings, further research is required to uncover the potential benefits of rehabilitation and exercise interventions in individuals with long COVID (97, 98). Concerns have been raised about the applicability of exercise-based treatments in those with long COVID due to the potential for post-exertional symptom exacerbation (29, 99, 100). For example, in a subset of individuals with long COVID, worsening of symptoms after physical or mental exertion has been reported (99, 100). In an observational study of 213 participants experiencing persistent symptoms due to COVID-19 that did not predate the confirmed or suspected infection, most individuals reported post exertional symptom exacerbation with 58.7% meeting the threshold for post exertional malaise (100). Therefore, exercise is not recommended for any individual with long COVID who experiences post exertional malaise or meeting diagnostic criteria for myalgic encephalomyelitis/chronic fatigue syndrome (ME/CFS). In such instances, other treatment strategies to manage energy levels such as “pacing” should be considered. Future studies examining for whom exercise is most appropriate, which symptom(s) should be targeted, and optimal dosing would be of great value to those living with long COVID.

## 8. Summary

SARS-CoV-2 causes multi-organ system dysfunction during the acute responses to infection. Delayed viral clearing resulting from immune dysfunction triggers chronic inflammation and the promotion of persistent symptoms of COVID-19. Lymphocyte activation and persistent inflammation has been noted in various settings post COVID, however, whether this persists long-term leading to constellation of signs and symptoms in long COVID needs to be investigated with urgency, so that clinicians are cognizant of these possible maladies. The combination of reduced *ACE2* and persistent inflammatory response may lead to chronic pulmonary vessel remodeling, though remodeling in other areas likely affects pulmonary function. While some cases of dyspnea may be due to direct damage to the pulmonary vasculature (even in the absence of pulmonary fibrosis), others may be due to increased venous capacitance resulting in the decreased preload with exercise. Progressive CKD in the setting of SARS-CoV-2 infection likely involves multiple mechanisms including persistent inflammation and maladaptive repair of injured nephrons from prior AKI. These mechanisms can come together potentially leading to interstitial fibrosis and glomerulosclerosis. Finally, exercise intolerance is a may have significant implications on functional capacity and quality of life in those with long COVID. Peripheral oxygen extraction is one potential factor contributing to the exercise intolerance experience by these individuals. While exercise may be beneficial for combatting the consequences of long COVID in some, the possibility of post-exertional symptom exacerbation highlights the importance for individualized treatment prescription. Despite the expansion of knowledge and understanding in long COVID, future research is needed to determine how this newly gained information can be best applied to maximize clinical benefit.

## Author contributions

SS contributed to the conceptualization of the manuscript. SS, SK, OA, SC, and JG wrote the first draft of the manuscript. SK, OA, SC, and JG wrote sections of the manuscript. SK and JG developed all figures for the manuscript. All authors contributed to the article and approved the submitted version.

## Funding

This work was supported in part by VA Career Development Award (CDA-2) (IKRX003423) and VA Small Projects in Rehabilitation Research (SPiRE) (I21RX004371) from the United States Department of Veterans Affairs, Office of Research and Development, Rehabilitation Research and Development Service (JG). This work was also supported by GW-Long COVID Grant Funds from George Washington University and Gut Microbiome-NAII Grant Funds (SS).

## Conflict of interest

The authors declare that the research was conducted in the absence of any commercial or financial relationships that could be construed as a potential conflict of interest.

## Publisher’s note

All claims expressed in this article are solely those of the authors and do not necessarily represent those of their affiliated organizations, or those of the publisher, the editors and the reviewers. Any product that may be evaluated in this article, or claim that may be made by its manufacturer, is not guaranteed or endorsed by the publisher.
